# A collaborative approach to improving representation in viral genomic surveillance

**DOI:** 10.1371/journal.pgph.0001935

**Published:** 2023-07-19

**Authors:** Paul Y. Kim, Audrey Y. Kim, Jamie J. Newman, Eleonora Cella, Thomas C. Bishop, Peter J. Huwe, Olga N. Uchakina, Robert J. McKallip, Vance L. Mack, Marnie P. Hill, Ifedayo Victor Ogungbe, Olawale Adeyinka, Samuel Jones, Gregory Ware, Jennifer Carroll, Jarrod F. Sawyer, Kenneth H. Densmore, Michael Foster, Lescia Valmond, John Thomas, Taj Azarian, Krista Queen, Jeremy P. Kamil

**Affiliations:** 1 Department of Biological Sciences, Grambling State University, Grambling, LA, United States of America; 2 School of Biological Sciences, Louisiana Tech University, Ruston, LA, United States of America; 3 Burnett School of Biomedical Sciences, University of Central Florida, Orlando, FL, United States of America; 4 Physics and Chemistry Programs, Louisiana Tech University, Ruston, LA, United States of America; 5 Mercer University School of Medicine, Macon, GA, United States of America; 6 Mercer Medicine, Macon, GA, United States of America; 7 Department of Chemistry, Jackson State University, Jackson, MS, United States of America; 8 Health Services Center, Jackson State University, Jackson, MS, United States of America; 9 Center of Excellence for Emerging Viral Threats, Louisiana State University Health Shreveport, Shreveport, LA, United States of America; 10 Department of Microbiology and Immunology, Louisiana State University Health Shreveport, Shreveport, LA, United States of America; CSIR-Indian Institute of Chemcial Technology, INDIA

## Abstract

The lack of routine viral genomic surveillance delayed the initial detection of SARS-CoV-2, allowing the virus to spread unfettered at the outset of the U.S. epidemic. Over subsequent months, poor surveillance enabled variants to emerge unnoticed. Against this backdrop, long-standing social and racial inequities have contributed to a greater burden of cases and deaths among minority groups. To begin to address these problems, we developed a new variant surveillance model geared toward building ‘next generation’ genome sequencing capacity at universities in or near rural areas and engaging the participation of their local communities. The resulting genomic surveillance network has generated more than 1,000 SARS-CoV-2 genomes to date, including the first confirmed case in northeast Louisiana of Omicron, and the first and sixth confirmed cases in Georgia of the emergent BA.2.75 and BQ.1.1 variants, respectively. In agreement with other studies, significantly higher viral gene copy numbers were observed in Delta variant samples compared to those from Omicron BA.1 variant infections, and lower copy numbers were seen in asymptomatic infections relative to symptomatic ones. Collectively, the results and outcomes from our collaborative work demonstrate that establishing genomic surveillance capacity at smaller academic institutions in rural areas and fostering relationships between academic teams and local health clinics represent a robust pathway to improve pandemic readiness.

## Introduction

In late 2019, a newly emergent betacoronavirus, severe acute respiratory syndrome coronavirus 2 (SARS-CoV-2), was identified as the etiological agent associated with an outbreak of viral pneumonia in Wuhan, China [1, 2]. The outbreak quickly grew to become a global public health emergency, now commonly referred to as the COVID-19 pandemic. Between November 2020 and January 2021, the first variants of concern (VOC) Alpha, Beta, and Gamma, were detected in England, South Africa, and Brazil and Japan, respectively. All three carried convergent spike (S) N501Y substitutions, while Beta and Gamma shared substitutions S:K417N/T and S:E484K. It is now appreciated that SARS-CoV-2 variants can be more transmissible [3], differ in pathogenicity [4, 5], and/or escape immunity afforded by vaccination or by infection with earlier variants [6, 7]. Although the Omicron lineage has displaced Delta [8], new Omicron sublineages continue to emerge [9], certain of which harbor concerning combinations of S substitutions that escape neutralizing antibodies, e.g., F486V, F486P, R346T, K444T, N460K, F490S [10–16]. After multiple waves of Omicron descendent lineages, the emergency phase of the pandemic has wound down, but COVID-19 remains largely uncontrolled in virtually all geographies. Despite a greatly reduced mortality rate, the virus remains a threat, particularly to older individuals, and moreover, continues to evolve [17]. Ongoing microbial genomic surveillance is necessary not only to promptly identify new SARS-CoV-2 variants, but also to detect and inform public health responses to other microbial threats, such as influenza viruses, monkeypox virus, countless pre-emergent bat-borne viruses, and antimicrobial resistance in bacteria [18–22].

Starting with the sharing on 10 Jan 2020 of the first “novel coronavirus 2019” genome [23], scientists have closely tracked the evolution of SARS-CoV-2. Viral genomic surveillance activities sprung up to sequence the genomes of circulating viruses, usually from patient nasal swabs, sharing their data via GISAID (www.gisaid.org) [24], and/or the international nucleotide sequence database collaboration (INSDC) [25]. These practices collectively enable scientists around the world to track viral evolution across space and time while keeping tabs on mutations that can compromise the utility of diagnostic assays or negatively impact the clinical efficacy of vaccines or monoclonal antibody therapeutics. Effective surveillance relies on prompt viral whole genome sequencing of recent samples across representative geographies and populations, as well as rapid, ‘low latency,’ sharing of resulting data. Accordingly, investments in viral whole genome sequencing have increased greatly during the pandemic, with vastly more whole genome sequences of SARS-CoV-2 being shared than any virus in history.

At the outset of the pandemic, SARS-CoV-2 genome sequencing efforts were patchy at best and in many geographies non-existent [26–28]. Although scientists in the U.K. and South Africa swiftly established exemplary viral genomic surveillance programs, in the U.S., SARS-CoV-2 genome data initially came in large part from ad hoc efforts that often relied on discretionary funds or support from private foundations and philanthropies [29–31]. The emergence in late 2020 of the first variants of concern prompted the U.S. Centers for Disease Control and Prevention and the National Institutes of Health to invest in the development of large-scale nationwide genomic surveillance. Despite this, many states in the Midwest and Southern U.S. remain poorly represented in the genomic surveillance data [32], even though their residents have been disproportionately impacted by COVID-19 [33–35]. This rural-urban disparity was compounded during the pandemic for racial and ethnic minorities [36, 37] who already bore a greater burden of disease [38]. The failure to adequately track emerging variants in a representative fashion may have exacerbated existing disparities in allocation of public health resources, such as rapid antigen tests and monoclonal antibody therapies.

**Fig 1:**
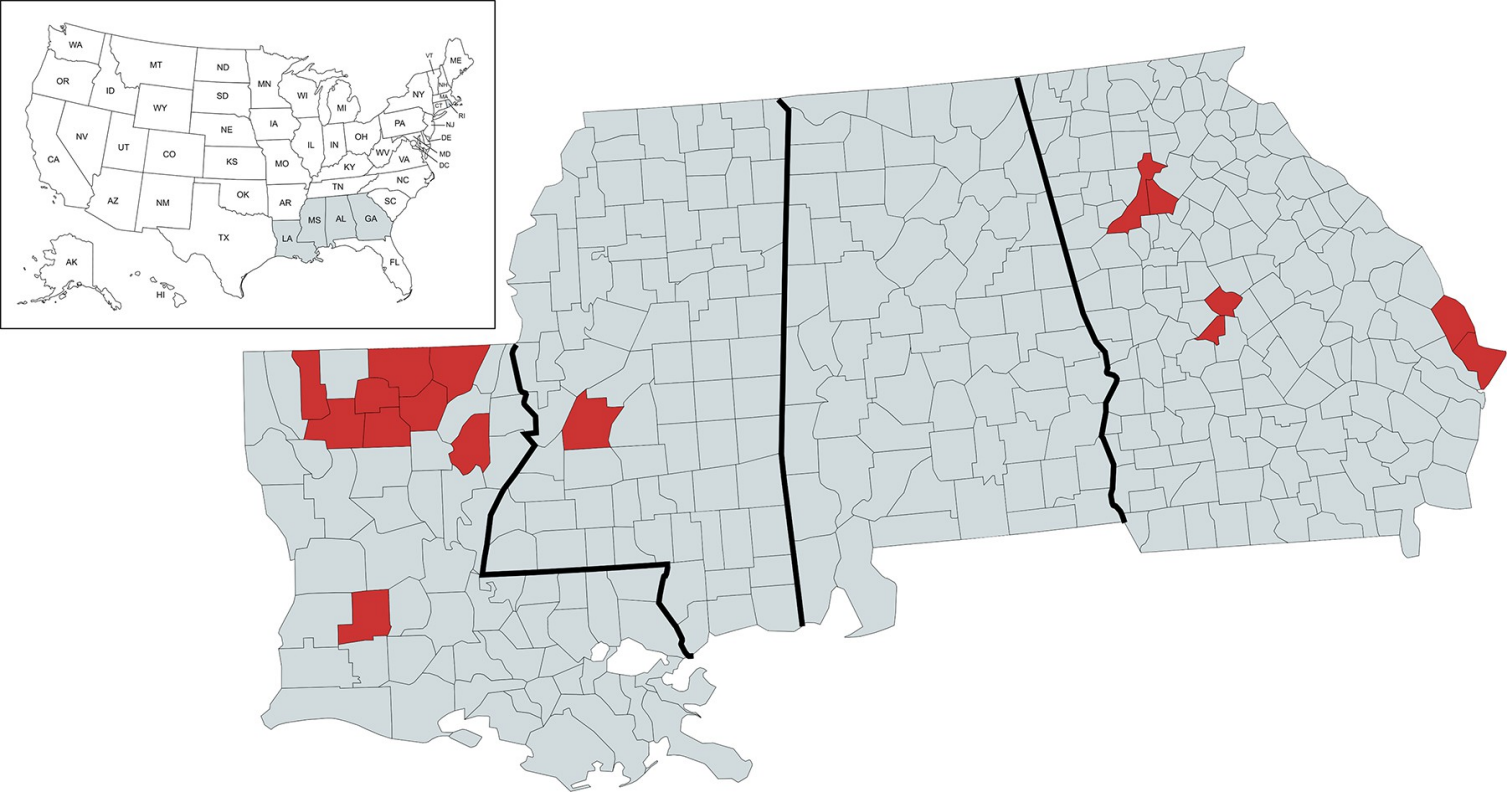
Geographic coverage of SARS-CoV-2 genomic surveillance in Louisiana, Georgia, and Mississippi. Map of the surveillance region with parishes (Louisiana) or counties (Georgia, Mississippi) where at least one specimen was sequenced by the network indicated in red. Map created using MapChart and republished under a CC BY license with permission from Minas Giannekas, original copyright 2014.

To address this lack of representation, Grambling State University, Louisiana Tech University, and Louisiana State University Health Shreveport established a viral genomic surveillance hub to reach several vulnerable and underserved populations in north Louisiana [39]. Soon after, we partnered with Mercer University School of Medicine in Georgia, Jackson State University in Mississippi, as well as with community health centers in each of our areas to increase SARS-CoV-2 genome sequencing volume and representativeness. These efforts resulted in a new viral genomic surveillance network serving a total of 16 counties or parishes across these 3 southern U.S. states (**Fig 1**).

Our primary goals were to improve sampling from rural communities and minorities, to empower underrepresented minorities conducting the scientific work of viral genomic surveillance, and to provide individuals who donate samples for viral genome sequencing the opportunity to do so with consent and an understanding of how their samples would be used to track viral variants. Most viral genomic surveillance programs sequence viral genomes from patient swabs under a ‘medical waste’ exemption, without obtaining the patient’s consent. In participatory surveillance projects, however, community members and volunteers collaborate to collect data, such as reporting weekly influenza symptoms [40]. Echoing this concept, at our project sites in Louisiana we educated patients about viral genomic surveillance and variant tracking, and provided them the opportunity to decide whether or not to donate their samples for sequencing.

The COVID-19 pandemic has also underscored the importance of contextual metadata accompanying genomic data. Incomplete metadata confounds comparative analyses and impedes data-driven policymaking [41, 42]. Thus, an additional objective was to collect more complete metadata to maximize the usefulness of the shared sequences. We used self-reported patient metadata to assess relationships between viral gene copies (as an index of viral load) and variant type, vaccination status, and symptomatology.

Here, we summarize the results of our work to improve representation and expand viral genomic surveillance in Louisiana, Georgia, and Mississippi.

## Results and discussion

### Improving representativeness in genomic surveillance

In Louisiana, our six partner clinics collected 405 rapid antigen-positive clinical specimens from the residents of nine parishes (Allen, Bienville, Franklin, Jackson, Lincoln, Morehouse, Ouachita, Union, and Webster) between 22 July 2021 and 12 April 2022 (**Fig 1**). We worked closely with clinicians at the collection sites to gain informed consent from patients and collected metadata on patient demographics and vaccination status for more than 90% of the specimens (**Table 1**).

**Table 1:** Characteristics of study donors in Louisiana (22 July 2021–12 April 2022).

CHARACTERISTICS	NUMBER OF DONORS (%)*
	n = 405
**Median age (range)**	22 (1–86) years
**Sex**	
Male	180 (44.4)
Female	215 (53.21)
Not disclosed	10 (2.5)
**Race/ethnicity**	
White	99 (24.4)
Black	205 (50.6)
Hispanic	34 (8.4)
Asian	4 (<1)
Pacific Islander	1 (<1)
Alaska Native	1 (<1)
Other	1(<1)
Not disclosed	66 (16.2)
**PCR**	
Negative	15 (3.7)
Positive	389 (96)
Indeterminate	1 (<1)
**Vaccination**	
No	209 (51.6)
Yes	170 (42)
Partial †	26
Full ‡	113
Booster §	18
Not specified ¶	13
Not disclosed	26 (6.4)
**Previous infection**	
No	321 (79.3)
Yes	41 (10.1)
Not disclosed	43 (10.6)
**Symptomatic**	
No	52 (12.8)
Yes	336 (83)
Not disclosed	17 (4.2)

*unless otherwise indicated

Vaccination

† one dose mRNA

‡ two dose mRNA or one dose Janssen

§ three dose mRNA

¶ specific type and date(s) of vaccination unknown

In these nine parishes, between 24.2% (Ouachita) to 82.9% (Union) of the residents live in rural areas as defined by the U.S. Census Bureau [43]. Rural areas may be especially vulnerable to the pandemic because they tend to have less capacity for diagnostic testing and healthcare services [44, 45] while being home to a poorer, older, health-compromised population [46]. Bienville and Franklin parishes in particular ranked among the 20 poorest counties out of 300 identified as having the highest COVID-19 mortality rates in recent analysis of 3,200 U.S. counties and county equivalents (e.g., parishes) [47]. Notably, the majority of the other parishes in the region were likewise characterized as low income and high mortality.

The disproportionate impact of COVID-19 on racial and ethnic minorities is well documented [48–50]. Nearly 60% of our donors self-identified as non-white or of mixed heritage (two or more races), compared to approximately 41% non-white overall in the sampled parishes per U.S. Census data [51]. Therefore, our data reflects enhanced representation of minority groups. In our view, this nominal oversampling nonetheless comports with equitable genomic surveillance because it compensates [52] for the overall disproportionately fewer samples from both rural and minority populations in the U.S. as a whole. Notably, 8.4% of our specimens were from Hispanic donors who make up just 3.1% of the northeast Louisiana population but were far more likely to contract COVID-19, accounting for 13.8% of overall cases in the region (up to 60% in some parishes) according to the Louisiana Department of Health COVID-19 dashboard [53]. Our partnership with The Health Hut mobile clinic, which was staffed with Spanish-speaking health care workers, contributed to improved representativeness among this population.

Overall, we detected SARS-CoV-2 RNA in 389 of the 405 specimens collected in Louisiana, using the U.S. CDC’s real-time RT-PCR diagnostic assay. We carried out whole viral genome sequencing on these PCR-positive samples, ultimately recovering viral genome data from 272 (70%) of them. These data represented 1.32% of the 20,617 genomes shared via GISAID from Louisiana during the reported period.

### Accelerating regional genomic surveillance

After successfully establishing relationships with local clinics and developing a pipeline for exchanging specimens and data across our network in Louisiana, we felt confident sharing our model with other peer institutions across the south. Mercer University School of Medicine in Georgia and Jackson State University in Mississippi both joined our efforts, building on relationships to engage their own communities in genomic surveillance.

In Georgia, COVID-19 positive patient swabs were collected by Mercer Medicine Student Health Services in Bibb, Chatham, DeKalb, Effingham, and Fulton counties and from a community clinic located in Peach County that is operated by Mercer Medicine (**Fig 1**). Roughly 81% of the Georgia specimens were obtained from counties outside of the Atlanta metropolitan area. Approximately 48% of Mercer donors did not self-identify as white. After testing positive via PCR analysis, specimens were sent to the Center of Excellence for Emerging Viral Threats (CEVT) at Louisiana State University Health Shreveport (LSUHS) for Illumina sequencing.

Among other achievements, our collaboration with Mercer identified two globally emergent Omicron sub-variants that were being monitored closely by the international community due to their concerning patterns of spike substitutions and rapid spread [54, 55]. These included the first documented case in Georgia and the 40th overall U.S. case of the BA.2.75 variant, as well as the sixth case in Georgia of BQ.1.1, one of the most antibody-evasive variants that had been described to date [54]. Overall, Mercer generated 703 SARS-CoV-2 genomes, representing 1.8% of the total data available in GISAID from Georgia between 13 September 2021, when the first Mercer sample was collected up to 10 October 2022.

At our Mississippi site, the Jackson State University (JSU) team made use of the JSU Health Services Center as its sample collection site (**Fig 1**). The JSU Health Services Center served as the primary COVID-19 testing site for students, staff, and visitors of the university and supported the surrounding community’s testing and vaccination efforts. The participation of JSU extended our project’s network into a predominantly black community that has long been subjected to environmental injustices and health disparities, including the recent collapse of its city water system [56]. One hundred percent of the 33 samples submitted for sequencing at the CEVT were from non-white donors.

### Building modern DNA sequencing capacity for local genomic surveillance

**Fig 2:**
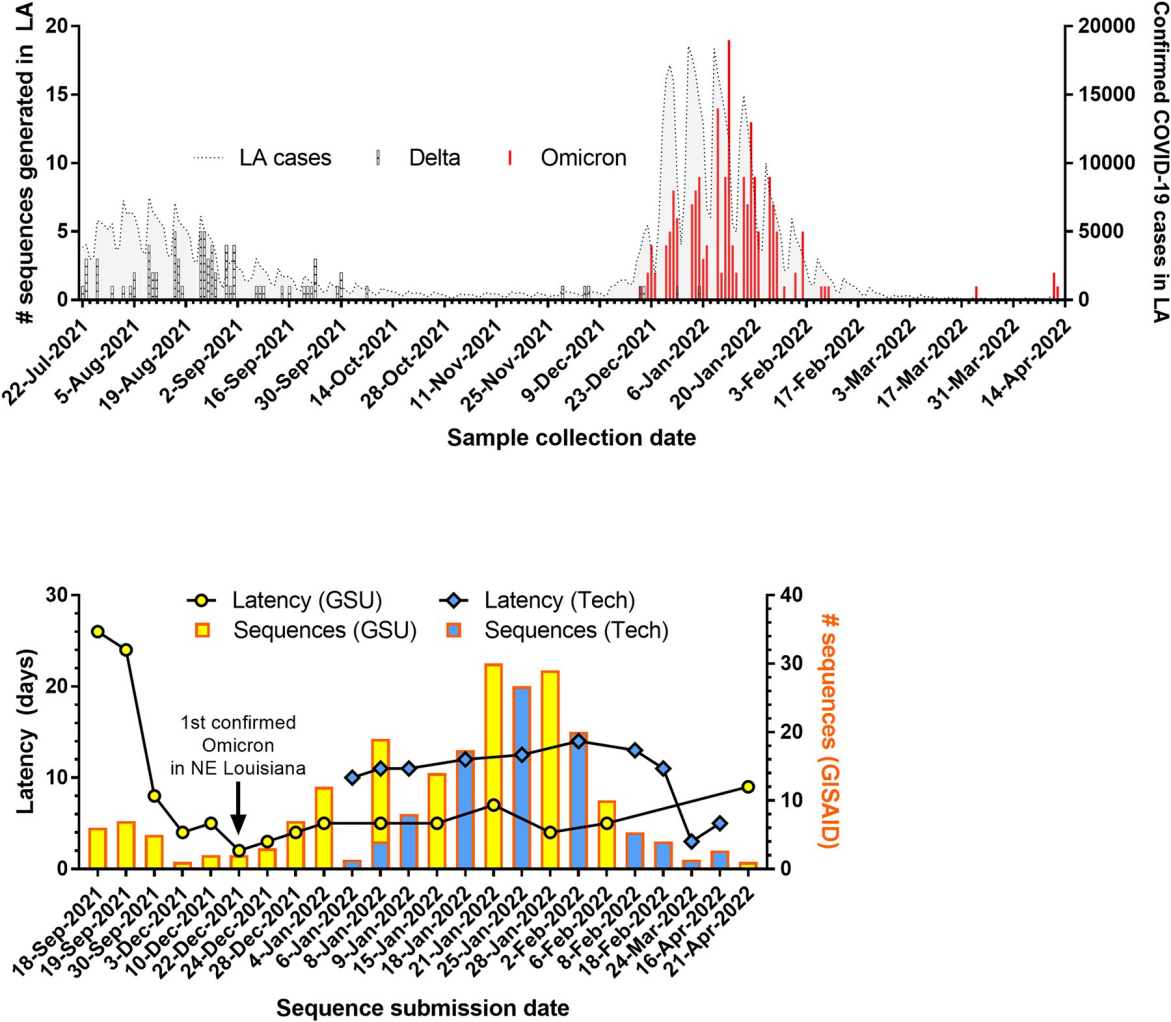
SARS-CoV-2 genomic surveillance in Louisiana. (A) Number of variants sequenced over time in Louisiana overlaid with number of confirmed COVID-19 cases in the U.S. (B) Number of sequences uploaded and latency for new sequencing entities established at Grambling State University (GSU) and Louisiana Tech University (Tech).

The specimens we collected in Louisiana were initially submitted to the LSUHS CEVT in Shreveport for sequencing until we built local sequencing capacity at Grambling State University (GSU) and Louisiana Tech (Tech). By sequencing locally, we improved our latency or turnaround time from sample collection to release on GISAID, from a median latency of 24 days to 5 days at GSU and 12 days at Tech when processing contemporary specimens (i.e., excluding results from archived samples) (**Fig 2B**). Through this approach, we identified the first confirmed case of Omicron in northeast Louisiana, a region comprising 12 parishes and 323,000 residents, within 2 days of sample collection [57]. We then shared the variant data along with weekly wastewater surveillance results and other COVID-19-related health information through a dashboard developed for the north Louisiana community (www.nla-health.com/dashboard).

The results of sequencing including investigation of viral genome coverage and copy number, and phylogenetic analyses are described below.

### Viral genome coverage

**Fig 3:**
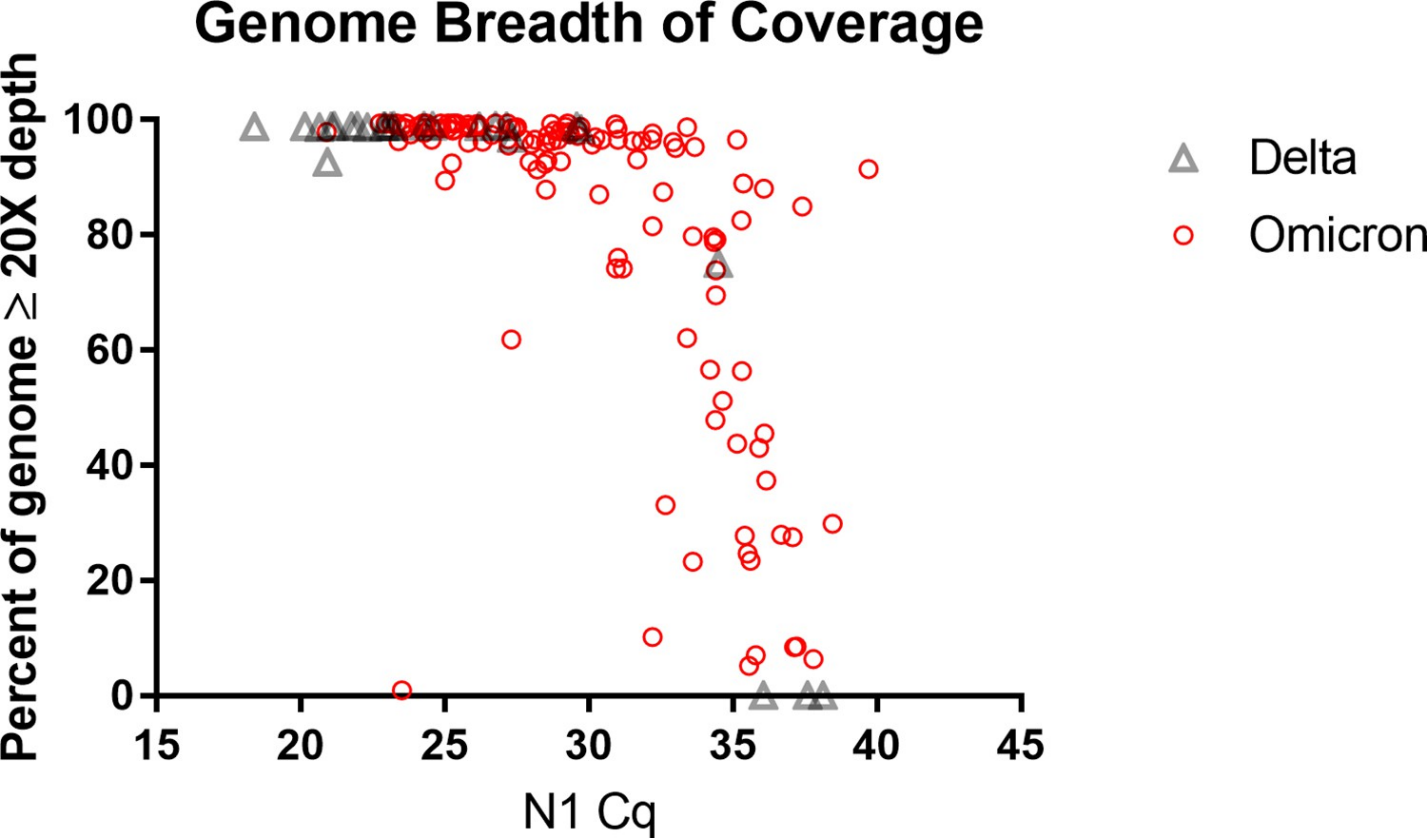
Decreasing genome breadth of coverage in samples with high Cq values. Higher Cq values were observed in Omicron (n = 115) than in Delta (n = 24). All but one Delta specimen was amplified for 32 cycles and all Omicron specimens were amplified for 35–40 cycles.

Plotting the breadth of coverage for Oxford Nanopore Technologies (Nanopore) sequencing reveals that samples with a reverse transcriptase quantitative PCR (RT-qPCR) threshold cycle (Ct) or quantification cycle (Cq) for viral RNA detection of approximately Cq 30 reliably resulted in complete genome coverage in our hands (**Fig 3**). In some cases, however, full viral genomes were recovered from samples with higher Cq values (indicative of fewer viral genomes in the starting material), and the upper limit in our setting was approximately Cq 35. These observations are congruent with what is seen in the literature across various ‘next generation sequencing’ (NGS) methodologies for SARS-CoV-2 genome sequencing [58]. Nonetheless, some groups using the Nanopore platform have reported high coverage consensus genome sequences from specimens with Cq values as high as 39 [59]. Variability in primer design and amplification parameters, library preparation method, and sequencing run time can influence results. For example, longer amplicons reduce the likelihood of primer interactions [60] and primer mismatches due to mutations [61], generally outperforming shorter amplicons [62, 63]. Shorter amplicons, however, can recover more complete genomes from specimens where the viral load is low or the RNA is degraded [64, 65].

Specimens with low viral loads can show uneven genome coverage owing to poor amplification of certain amplicons. Such coverage problems can be overcome to some extent by optimizing PCR and sequencing protocols. For instance, Lagerborg *et al*. found that increasing PCR cycles from 35 to 40 resulted in more uniform coverage for Illumina-based ARTIC sequencing of low viral load specimens while still avoiding PCR artifacts [65]. Compared to the Nanopore rapid barcoding kit used for library preparation in our study, the more labor-intensive Nanopore ligation sequencing kit can yield greater sequencing depth and better (*de novo*) assembly of the SARS-CoV-2 genome [66]. Of course, longer run times can also increase the number of mapped reads to enhance recovery of complete genomes.

### Viral gene copies

The fast-spreading Omicron variant swiftly displaced the Delta variant in our sampling, with a minimal period of overlap (**Fig 2A**). We find that the mean Cq value of Omicron specimens (28.87, 95% CI 28.13–29.61) was significantly higher (p < 0.0001) than that of Delta specimens (24.09, 95% CI 22.51–25.66). This corroborates early reports of lower viral gene copy number determined by RT-qPCR [67, 68] or lower infectious viral load determined by focus forming assay [69] in Omicron infections compared to Delta infections.

One possible explanation for the observed difference in gene copy number/imputed viral load is that during the initial Omicron surge, a greater proportion of the population may have acquired some degree of immunity through vaccination or prior infection. Some reports support the idea of attenuated viral load with vaccination [70–72], while others found no significant difference in viral genome copies between unvaccinated and vaccinated individuals [73–75].

**Fig 4:**
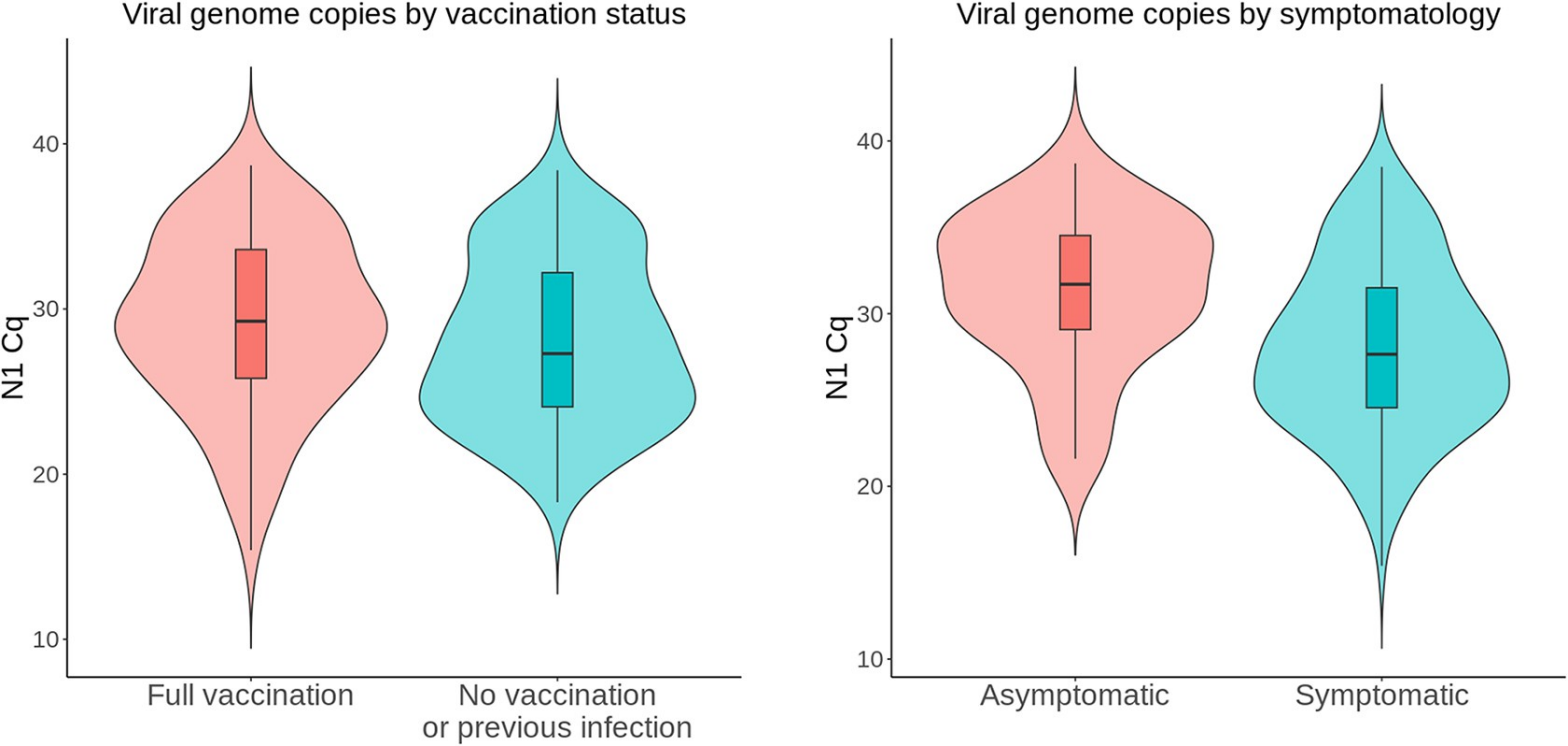
Viral gene copies by vaccination status and symptomatology. (A) No statistically significant differences (p = 0.1191) in viral gene copies were observed between unvaccinated donors without a previous infection (n = 80) and vaccinated donors with or without a previous infection (n = 80). (B) Viral gene copies were lower (p = 0.0006) in asymptomatic donors (n = 30) compared to symptomatic donors (n = 160).

Having collected vaccination and prior infection metadata from 379 and 362 of the 405 donors respectively, we compared immune history with viral gene copy numbers. We found no statistically significant differences in the mean Cq value between unvaccinated individuals with no previous infection (n = 80) and fully vaccinated individuals with or without a previous infection (n = 80) (**Fig 4A**).

Several groups have found no correlation between RT-qPCR quantification of viral genes and symptomatology [76–79]. Two studies reported higher Cq values in asymptomatic individuals (n = 5 or 3) compared to symptomatic individuals (n = 14 or 9) but the sample sizes were limited [80, 81]. In a larger study, asymptomatic individuals (n = 2179) exhibited higher Cq values compared to symptomatic individuals (n = 739); however, the authors concluded that the statistically significant difference of 0.71 cycles was not clinically meaningful [82]. In our dataset, the mean Cq value of asymptomatic donors (31.36, 95% CI 29.7–33.01; n = 30) was more than 3 cycles higher (p = 0.0006) than that of symptomatic donors (28.04, 95% CI 27.28–28.8; n = 160) (**Fig 4B**).

The asymptomatic donors in this study were mostly college students who tested positive during baseline surveillance on the GSU campus, so the age distribution is highly skewed compared to symptomatic donors. There are conflicting reports on the relationship between patient age and viral load, but one of the largest cross-sectional studies found that viral load tends to increase with age [83]. To account for a possible age effect, we compared asymptomatic donors who were age 30 or younger (n = 28) with symptomatic donors in the same age group (n = 93) and found that the asymptomatic group still had lower (p = 0.0057) mean viral gene copy number (31.78, 95% CI 30.17–33.39 vs 28.55, 95% CI 27.39–29.7).

Only Cq values derived at GSU were used in these analyses to avoid potential confounding by interlaboratory variation in PCR assays [84]. These analyses should be considered carefully within the limitations of our study which include a relatively small sample size and lack of longitudinal information on peak copy number, and also within the overarching context that copy number is an imprecise index of viral load [85].

### Phylogenetic analysis of Louisiana and Georgia SARS-CoV-2 genomes

**Fig 5:**
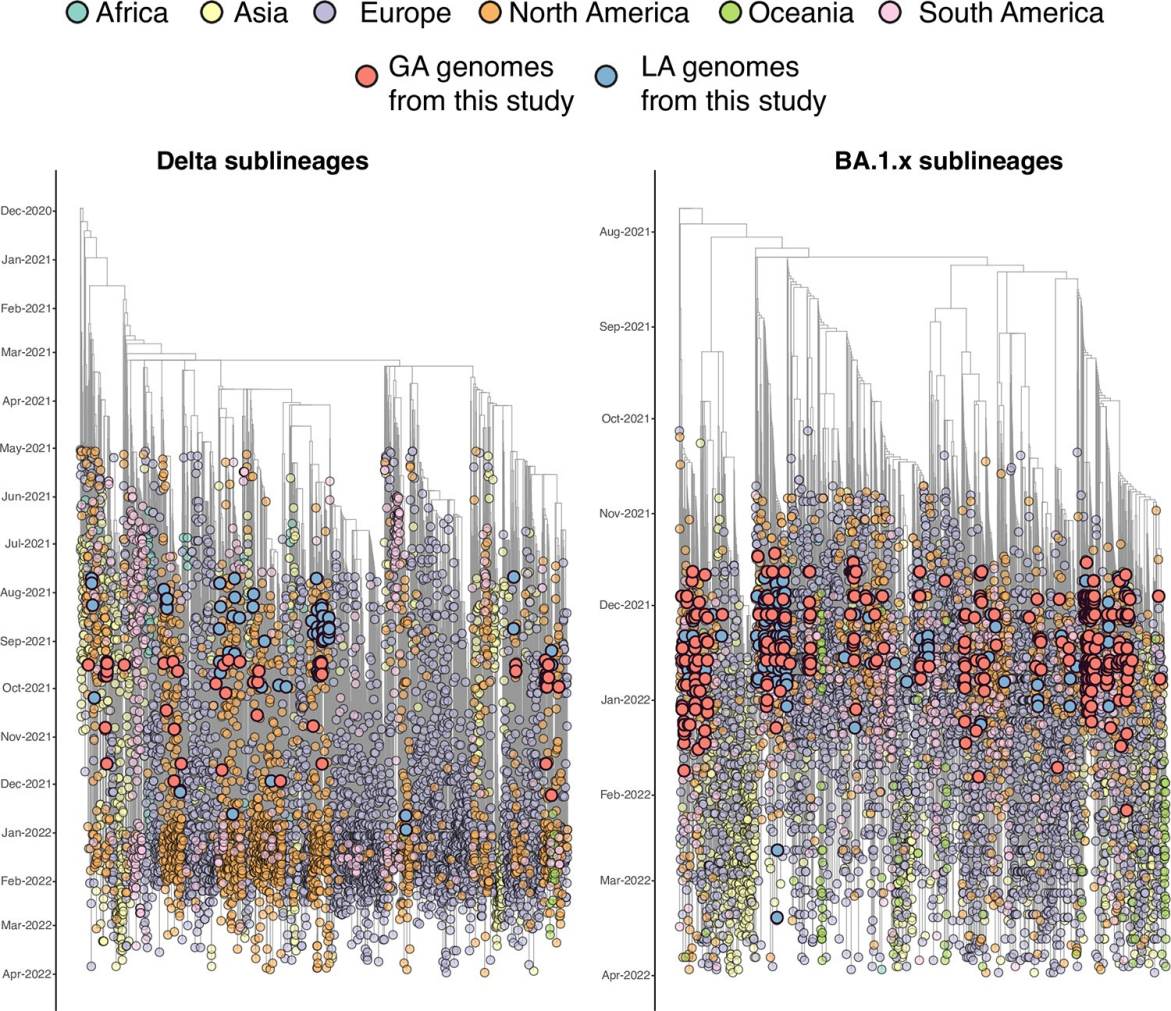
Time-resolved Maximum Likelihood (ML) phylogenies of Delta and Omicron (BA.1.x) sublineages. The genomes generated in this study are colored in red for Georgia and blue for Louisiana. Tree tips are colored according to their location (continent) and the color legend is on the top of the figure.

We performed independent phylogenetic analysis for Delta and BA.1.x SARS-CoV-2 viral populations from Louisiana and Georgia in the context of representative global samples. Time-scaled maximum likelihood phylogenies revealed that Delta and BA.1.x isolates from Louisiana and Georgia are interspersed throughout the global population suggesting multiple independent introductions (**Fig 5**). Often, these introductions resolve into distinct subclades indicative of ongoing transmission within a new location. Further, putative introductions were spatially diverse and included Europe and South America as well as other states throughout the US. These local epidemics were nested within the larger North American epidemic for both Delta and BA.1.x waves.

## Conclusion

### Collaborative community-centered genomic surveillance networks

As part of a broader initiative supported by the Rockefeller Foundation’s Pandemic Prevention Institute, we established a genomic surveillance network focused on equitable viral genomic surveillance among more vulnerable and marginalized populations. To achieve this, we formed collaborations between institutions with very high research activity, primarily undergraduate universities, a graduate medical school, Historically Black Colleges and Universities, and local community health centers. Through this collaborative and decentralized approach, we sequenced viral genomes from respiratory specimens provided by patients in rural areas of Louisiana, Georgia, and Mississippi. These three states rank 41^st^, 43^rd^, and 45^th^ respectively among U.S. states in the percentage of cumulative cases sequenced [32]. Thus, with only a modest investment from the Rockefeller Foundation and internal support from our institutions, we increased the viral genomic surveillance coverage from these states such that the sequenced samples better represented the ethnic, racial, and cultural diversity of the U.S. population. Our approach also enabled us to collect and share rich metadata, including vaccination status and county/parish-level geographical location, which is particularly important when emerging variants such as BA.2.75 and BQ.1.1 are detected. In both the GSU and Tech initiatives, health care workers at partnering clinics briefly educated patients about how viral genomic surveillance is used to track emerging variants and provided patients with the opportunity to provide consent for their positive sample to be used for whole viral genome sequencing.

Residents of rural areas face significant barriers rooted in geographic isolation, scarcity of services, lower socioeconomic status and other social determinants of health, and even cultural constraints that impede them from getting the healthcare that they need [86]. During the pandemic, this disparity manifested as disproportionately high mortality rates in rural America, particularly among minorities [87]. In rural areas, so-called regional universities represent essential infrastructure that can provide not only educational opportunities but also access to testing and vaccination. These universities are uniquely positioned to gain trust and encourage participation in genomic surveillance because they often function as community anchors, vital to the local economy, civics, and culture [88]. A viral genomic surveillance strategy that leverages these strong community ties also offers a much-needed on-ramp for students from diverse groups to gain genomics research experience that will prepare them to combat misinformation in their own communities and participate in the genomics workforce.

### Viral genomic surveillance at all scales

Oxford Nanopore Technologies (Nanopore) DNA sequencing has been used for real-time genomic surveillance during outbreaks of Ebola in West Africa [89], Zika [90] and Yellow Fever [91] in Brazil, and Lassa fever in Nigeria [92]. During the COVID-19 pandemic, many laboratories around the world used Nanopore sequencing to rapidly generate and share SARS-CoV-2 genome data. The Nanopore platform is especially suitable for smaller institutions because start-up costs and infrastructure requirements are minimal, operational costs are low, and the approach is highly scalable. A modestly equipped molecular biology laboratory can implement a Nanopore rapid barcoding “swab to data” workflow for less than $3500 USD at current prices. Operational costs, excluding RNA extraction, are estimated at £18.91 ($23.61 USD) per ligated barcode for 12 barcodes on a MinION flow cell when using a wash kit to remove the previous library [93]. This corresponds well with our cost analysis, which *includes* RNA extraction, of $20 –$25 USD per tagmented barcode for 12 barcodes when the flow cell was washed and reused.

Working with raw genome sequencing data often requires significant computational resources and bioinformatics expertise. However, Nanopore-based viral genome sequencing can be performed on a consumer-grade personal computer, using readily available workflows that include cloud-based, “*point-and-click*” assembly and analysis pipelines. Although higher throughput facilities can multiplex 96 or more barcodes at a cost of less than £10 ($12.50 USD) per barcode [93], the platform enables smaller laboratories to cost-effectively provide genomic surveillance on limited numbers of samples, thus enhancing the ability reach underserved communities and provide much needed coverage in under-sampled areas.

The democratization of pathogen genomics is nowhere better demonstrated than in Africa, where technological advances in viral genome sequencing have been coupled with major investments by Africa CDC and its partners who rapidly expanded genomic surveillance across the continent [94]. These efforts in Africa resonate with some of our experiences in the rural Southern U.S., highlighting that locally based programs can close blind spots in viral genomic surveillance while significantly decreasing turnaround time. The success of these types of decentralized, community-based approaches is exemplified by our identification of the first confirmed case of Omicron (B.1.1.529, or ‘BA.1’) in northeast Louisiana, and by our early detection in Georgia of concerning emergent Omicron sublineages BA.2.75 and BQ.1.1.

Overall, our work demonstrates the positive impact that universities can have within their communities, in particular the power of partnerships forged between academic teams and local community clinics to build engagement and foster participation while generating valuable data relevant to public health responses. We argue that policymakers and funding agencies should strongly support the formation of these types of nimble, local networks in global strategies to modernize viral genomic surveillance of common respiratory illnesses. To facilitate the expansion of community-based genomic surveillance programs, we have developed a guide entitled, *Building a Collaborative and Equitable Viral Genomic Surveillance Program*: *A Playbook for Researchers*, *Clinicians*, *Administrators*, *and Allies*, that offers a modular and flexible roadmap for universities and local clinics to set up their own viral genomic surveillance program (please contact the corresponding authors for information). When community-based programs that rapidly generate and share pathogen genome data become commonplace across geographies, the world will be better poised not only to promptly detect new pandemic threats but also to train a diverse and representative public health workforce for tomorrow.

## Methods

### Specimen collection

Patients testing positive (*n* = 405) on the BinaxNOW rapid antigen test (Abbott Laboratories; Abbott Park, IL) at the Foster Johnson Health Center in Grambling State University (Grambling, LA), The Health Hut (Ruston, LA), Louisiana Tech University (Ruston, LA), Michael Brooks Family Clinic (Ruston, LA), Minden Family Care Center (Minden, LA), and Serenity Springs Specialty Hospital (Ruston, LA) between 23 August 2021 and 12 April 2022 were recruited through an informed consent process. Remnant anterior nasal swabs used in the rapid test (The Health Hut, Michael Brooks Family Clinic, Louisiana Tech University, Minden Family Care Center, Serenity Springs Specialty Hospital) or freshly collected mid-turbinate nasal swabs (Foster Johnson Health Center) were preserved in 3 mL of viral transport media (VTM) at 4°C and later in the study in 1 mL of DNA/RNA Shield to inactivate the virus and allow for storage at room temperature.

### Ethical considerations

Specimen collection in Louisiana clinics was conducted under 45 CFR 46.102(l)(2) as a public health surveillance activity. Consent was obtained in writing from all participants in Louisiana in accordance with Louisiana Tech University (Ruston, LA) IRB protocol HUC 21–106. Specimen collection in Georgia was conducted under Mercer University (Macon, GA) IRB protocol H2110206. Specimen collection in Mississippi was conducted under Jackson State University (Jackson, MS) IRB protocol 0097–22. Consent was not obtained from participants in Georgia or Mississippi because the IRBs only approved collection of fully de-identified clinical remnant samples. Sequencing of samples at LSUHS CEVT was conducted under IRB protocol # STUDY00001445.

### Illumina sequencing (LSUHS CEVT)

Total RNA was isolated from VTM within 72 hours of specimen collection using the Omega Bio-Tek Viral RNA Xpress kit (Norcross, GA) on an automated extraction platform. Presence of SARS-CoV-2 RNA was determined by using CDC primers and probes with LunaScript RT Supermix Kit (NEB) run on BioRad (Hercules, CA) C1000 real-time PCR machines. cDNA, PCR amplification, and library preparation was performed using the NEBNext ARTIC SARS-CoV-2 FS kit (NEB) according to the manufacturer’s instructions. Libraries were quantified using Invitrogen Qubit BR dsDNA kit and size determined on an Agilent Tapestation 2200 using high sensitivity d1000 tapes (Santa Clara, CA). Libraries were loaded at 10.5 pM on an Illumina (San Diego, CA) MiSeq version 2, 300 cycle kit on an Illumina MiSeq instrument. Consensus sequences were generated with an in-house data analysis pipeline using bwa, samtools, and ivar.

### Nanopore sequencing (GSU and Tech)

Total RNA was extracted from VTM or DNA/RNA Shield within 48 hours of specimen collection via the QIAmp Viral RNA Minikit (Qiagen; Germantown, MD) or Zymo Quick-DNA/RNA Viral MagBead Kit (Zymo Research; Irvine, CA) according to the manufacturers’ protocols. Briefly, 500 uL of VTM was centrifuged at 5,000 g for 10 minutes to pellet cells and debris and either 140 uL of supernatant was extracted in 560 uL of Buffer AVL containing 560 ug of carrier RNA (QIAmp) or 200 uL of supernatant was extracted in 400 uL of Viral DNA/RNA Buffer followed by addition of 10 uL MagBinding Beads (Zymo). After washing the spin column or beads with the manufacturers’ respective wash buffers, total RNA was eluted from the column in 60 uL of Buffer AVE (QIAmp) or from the beads in 30 uL of DNase/RNase-free water (Zymo) and immediately used for RT-qPCR and library preparation. The presence of SARS-CoV-2 was confirmed using the SARS-CoV-2 Research Use Only qPCR Primer and Probe Kit (Integrated DNA Technologies, IDT; Coralville, IA) and qScript XLT One-Step RT-qPCR ToughMix (Quantabio; Beverly, MA) according to the CDC 2019-nCoV Real-Time RT-PCR Diagnostic Panel procedure.

The sequencing library was prepared using the 1200 bp amplicon "midnight" primer set (versions 1 or 2) [95] following the protocol by Freed and Silander [61]. Briefly, the RNA was reverse transcribed using the LunaScript RT Supermix Kit (New England BioLabs, NEB; Ipswich, MA). Tiled 1200 bp amplicons were generated using midnight primers (IDT) with Q5 Hot Start High-Fidelity 2X Master Mix (NEB) for 32 cycles (LA-GSU1 to LA-GSU19) or up to 40 cycles (LA-GSU20 to LA-GSU148 and LA-TECH1 to LA-TECH68) of multiplex PCR amplification. The two overlapping amplicon pools for each specimen were combined and quantified using the Qubit dsDNA HS Assay Kit (Invitrogen; Carlsbad, CA). A total of 75–150 ng of PCR product per specimen was barcoded using the Nanopore Rapid Barcoding Kits SQK-RBK004 or SQK-RBK110.96 (Oxford Nanopore Technologies, ONT; Oxford, UK). The barcoded samples were pooled and purified using AMPure XP beads at a 1:1 ratio of sample:beads (Beckman Coulter; Brea, CA). The final libraries (350–800 ng DNA) were sequenced on an R9.4.1 flow cell using a MinION Mk1B or Mk1C device, basecalled in real-time via MinKNOW, and analyzed using the wf-artic workflow (medaka, minimap2, bcftools, samtools, nextclade, artic, pangolin) according to the ONT protocol.

### Nanopore concordance with Illumina

We validated the performance of Nanopore MinION sequencing at GSU and Tech by sequencing a matched subset of 16 SARS-CoV-2 positive specimens using a well-established Illumina workflow at the CEVT. The results confirmed the accuracy of the Nanopore sequencing protocol and analysis pipeline in our setting with 100% consensus sequence identity in 13/16 of the samples and 99.9% sequence identity in 3/16 samples. The one discordant basecall was identical in the three samples: T by Illumina sequencing and G by Nanopore sequencing at 17,259, within the gene encoding the replicase accessory protein NSP13. The reference SARS-CoV-2 genome also contains a G at this position; the T mutation identified by Illumina sequencing results in a glutamic acid rather than an aspartic acid. The E1264D mutation identified in the samples sequenced with the Illumina protocol is well supported. Nanopore basecalling accuracy decreases in homopolymeric tracts [96], but there was not a homopolymer in this region, so the reason for this discordance remains unclear.

Importantly, all sequences were 100% concordant for the Pango lineages and GISAID clade assignments. The 16 samples sequenced by both protocols spanned a wide range of Cq values (15.6–32.4) but almost all samples, Nanopore or Illumina, generated > 98% genome coverage (Nanopore: 98.73%, Illumina: 99.67%). Illumina sequencing resulted in an average of 285 bases more sequence data than Nanopore, but this was mainly confined to the extreme 5’ and 3’ ends of the genome.

### Phylogenetic analysis

SARS-CoV-2 genomes were downloaded from GISAID (www.gisaid.org) excluding the low coverage and incomplete records up to 30 April 2022, and subsampling carried out using subsampler to obtain a representative dataset [97]. Subsampling 4,199,772 (Delta sublineages) and 2,895,902 (BA.1.x sublineages) genomic sequences resulted in 5,882 (Delta sublineages) and 6,843 (BA.1.x sublineages) total sequences. Viral sequences were aligned using ViralMSA with default parameters [98] using Wuhan-1 (MN908947.3) reference and then manually curated with Aliview to designate the start site and trim terminal regions [99]. A maximum likelihood (ML) phylogeny was inferred using IQTREE v1.6.10 with the best-fit nucleotide substitution model identified by the Model Finder function [100]. Statistical support for nodes was determined using 1,000 bootstrap replicates. TreeTime [101] was used to transform this ML tree topologies into a dated tree using a constant mean rate of 8.0 × 10^−4^ nucleotide substitutions per site per year, after the exclusion of outlier sequences. The phylogeny was visualized using the *ggtree* package in RStudio with tips colored by collection source (continent).

### Statistical analysis

Statistical analysis was performed using Prism version 7.05 (GraphPad; San Diego, CA). Continuous variables between two groups were tested for normal distribution (D’Agostino & Pearson test) and homogeneity of variance (F test) then compared using an independent t-test with two-tails and alpha set to 0.05.

## Supporting information

S1 Table EPI_SET ID for sequences from this initiative. (PDF)

S2 Table EPI_SET ID for subsampled set of global sequences. (PDF)
