# Supplementary material for: A collaborative approach to improving representation in viral genomic surveillance
Source: PLOS Glob Public Health. 2023 Jul 19;3(7):e0001935. doi: 10.1371/journal.pgph.0001935 (PMC10355392; doi:10.1371/journal.pgph.0001935)
Supplement: S2 Table — (PDF) [file pgph.0001935.s002.pdf]

SUPPLEMENTAL TABLE

Data Availability

GISAID Identifier:	EPI_SET_220901cu
doi:	10.55876/gis8.220901cu

All genome sequences and associated metadata in this dataset are published in GISAID's EpiCoV database. To view the contributors of each individual sequence with details such as accession number, Virus name, Collection date, Originating Lab and Submitting Lab and the list of Authors, visit 10.55876/gis8.220901cu

Data Snapshot

- EPI_SET_220901cu is composed of 13,409 individual genome sequences.
- The collection dates range from 2021-05-02 to 2022-04-30;
- Data were collected in 78 countries and territories;
- All sequences in this dataset are compared relative to hCoV-19/Wuhan/WIV04/2019 (WIV04), the official reference sequence employed by GISAID (EPI_ISL_402124). Learn more at https://gisaid.org/WIV04.
